# Does participation in the European Trauma Course lead to new behaviours and organisational change? A Portuguese experience

**DOI:** 10.1186/s12909-023-04322-0

**Published:** 2023-06-06

**Authors:** Elizabete Neutel, Sebastian Kuhn, Peter Driscoll, Carl Gwinnutt, Zélia Moreira, Ana Veloso, Maria Conceição Manso, António Carneiro

**Affiliations:** 1 Department of Anaesthesiology, Intensive Care Medicine and Emergency, Porto University Hospital: Centro Hospitalar Universitário de Santo António (CHUd SA), Largo Professor Abel Salazar, 4099-001 Porto, Portugal; 2 Institute of Digital Medicine, Philipps-University Marburg and University Hospital of Giessen and Marburg, Marburg, Germany; 3 Faculty of Clinical and Biomedical Sciences, University of Central Lancashire, Preston, PR1 2HE UK; 4 Resuscitation Council UK, Tavistock Square, London, WC1H 9HR UK; 5 CICS. NOVA. UMinho; School of Psychology, University of Minho, 4704-553 Braga, Portugal; 6 Faculty of Health Sciences, FP-I3ID/FP-BHS, University Fernando Pessoa, 4200-150 Porto, Portugal; 7 Hospital da Luz Arrábida, 4400-346 Vila Nova de Gaia, Portugal

**Keywords:** ETC, Education, Trauma, Holton`s evaluation model, Kirkpatrick’s hierarchy model, Learning transfer system inventory, Utstein formula of survival

## Abstract

**Background:**

Medical educational courses can be successful from an immediate feedback perspective but not lead to new behaviour or organisational changes in the workplace. The aim of this study was to assess the self-perceived impact of the European Trauma Course (ETC) on Reanima trainees’ behaviour and organisational change.

**Methods:**

A 40-item questionnaire based on Holton's evaluation model was used to evaluate the candidate's perceptions. The results were analysed with descriptive and inferential statistical analysis using nonparametric tests with α = 0.05.

**Results:**

Out of 295 participants, 126 responded to the survey. Of these, 94% affirmed that the ETC modified their approach to trauma patients, and 71.4% described a change in their behaviour. Postcourse responders changed their behaviour in their initial approach to trauma care in the nontechnical skills of communication, prioritisation and teamwork. Being an ETC instructor strongly influenced the acquisition of new material, and this group was able to implement changes in attitudes. Individuals with no previous trauma course experience identified lack of self-efficacy as a significant obstacle to introducing new work-based learning. In contrast, responders with ATLS training noted a lack of ETC colleagues as the main impediment for moving from conceptualisation to experimentation in the workplace.

**Conclusions:**

Participation in the ETC led to behavioural changes in the workplace. However, the ability to influence others and bring about wider organisational changes was more difficult to achieve. Major factors were the status of the person, their experience and self-efficacy. National organisational impact was obtained, which went far beyond our aspirations in acknowledging change in individual daily practice.

Future research studies will include the effect of implementing the ETC methodology on the outcome of trauma patients.

**Supplementary Information:**

The online version contains supplementary material available at 10.1186/s12909-023-04322-0.

## Background

The European Trauma Course (ETC) is an evidence-based trauma management course for the early in-hospital phase of delivering trauma care. In addition to direct trauma resuscitation training, it also teaches team leadership, team membership and team management [1]. This is achieved by using a team-based practical approach that includes hybrid simulation that allows for local adaptations to meet regional European needs [1–4].

Reanima is a Portuguese nonprofit organisation that teaches critical care medicine (CCM), resuscitation and trauma management. It has participated in the ETC since its inception and has run twenty courses between 2015 and 2021.

This ETC study, organised by Reanima, was designed to blend qualitative and quantitative approaches in a framework of evaluative enquiry. Kirkpatrick hierarchy four-level evaluation model [5] is a widely used tool for evaluating medical training interventions but, there are accepted limitations. Reanima uses this model to evaluate first level results of training courses, obtaining important information to the training organisers. However, Kirkpatrick approach it is not so efficient in evaluating the knowledge transfer to the workplace because there is evidence that there are other variables involved in the knowledge transference. In our study we proposed the Holton's evaluation model with a more comprehensive framework for diagnosing and understanding the causal influences of Human Resource Development (HRD) intervention outcomes. Three outcome levels are assumed in this model: learning, individual performance and organisational performance. Outcomes can be influenced by primary intervening variables (e.g., ability, motivation to learn, reaction to learning, transfer conditions, expected utility, linkage to organisational objectives) and secondary intervening variables (e.g., job attitudes, personality characteristics) [6].

Assessment of the ETC, organised by Reanima, has been based on postcourse evaluations by candidates, national faculty and audits by international faculty. These have all been positive, and trainees have frequently indicated a high degree of satisfaction with changes in all three domains of learning (knowledge, skills and affect). Studies have also shown evidence of knowledge acquisitions via pre- and postevaluation, with similar findings in literature reviews [7–9]. However, there are accepted limitations to this form of evaluation. Subjective assessment represents the lowest level of Kirkpatrick’s hierarchy [5], and previous studies have shown that there is little or no relationship between a person’s experiential learning and the ability to transfer the new knowledge into their daily practice [10, 11]. Therefore it remains to be clarified whether trainees transfer their ETC experiential learning into the workplace.

The aim of the present study was to assess the self-perceived impact of the ETC on Reanima trainees’ behaviour and organisational change. To date, we are unaware of any other studies that have provided an analysis of ETC impact on trainees’ transfer of experiential learning and the mechanisms of implementation into their daily practice, taking into account the factors that hinder or facilitate knowledge transfer.

## Methods

### Study participants and setting

Reanima organised sixteen ETC courses between February 2015 and June 2019, with 295 participants from all regions of Portugal and different trauma realities. The number of participants per edition ranged from 12 to 24 candidates. The candidates gain access to ETC by paying the course themselves or were sponsored by their employer.

### Intervention

ETC is an evidence-based trauma management course for the early in-hospital phase of delivering trauma care. It is a course with trauma scenarios simulations (30 trauma scenarios and 10 workshops) and 2 lectures covering all aspects of major trauma. One of the fundamental ETC points in trauma resuscitation is the importance of team horizontal approach to the primary survey. ETC reflects European practices, being flexible to reproduce local practices too. The TraumaTeam Members (TTM) include an Airway(A), Breathing(B), Circulation (C) person and a Trauma Team Leader (TTL).

The non-technical skills (NTS) are key areas developed in ETC and they are essential for team`s effectiveness, all of which are closely linked and integrated by communication. NTS are, in this context: team work (e.g., working together with a common goal, being accountable for your actions), task management (e.g., the Team Leader must ensure: A, B, C tasks according to team members’ competencies), situational awareness (e.g., knowing what is going on around you), decision making (e.g., prioritize, encourage team input) and communication (e.g., all key findings are clear to everyone, use of person´s names, closed loop communication).

Assessment on ETC is done continuously during the 1^st^ & 2^nd^ day (technical skills, team membership skills, team leadership skills) and summative assessment on the 3^rd^ day (team leadership skills).

### Data collection

A questionnaire based on Holton’s evaluation model [6, 12] was used to assess whether ETC leads to behavioural changes on the Reanima trainees’ workplace (Google^©^ form), i.e., is evaluated not only the individual motivation to transfer but also a favourable organisational climate to transfer. Initially created by a Reanima ETC faculty working group, it was modified in light of feedback provided by senior faculty participants. The survey was based on voluntary participation and information disclosure. The study conformed with the principles of The Declaration of Helsinki, and participation was taken as consent. All volunteers were allowed to withdraw from the survey at any time during data collection without any prejudice. Participant data were treated in line with the General Data Protection Regulation. Regarding the study data and analyses, all identifying data were removed. Moreover, no data analysis was performed that allowed for the publication of results that could eventually indirectly identify a participant. The survey was carried out according to the formal approval of the Research Center of Centro Hospitalar Universitário de Santo António.

Forty questions covered six topics and are accessible in Additional file 1:

- Respondents’ biographic data (e.g., age, sex, professional category, workplace, previous experience teaching CCM or trauma)
- Motivation to attend an ETC course
- Previous trauma training
- Trauma experience in the workplace, including a role as a trauma team leader (TTL) or trauma team member (TTM)
- Workplace changes to the management of major trauma patients, including team approach, communication; acquisition of new material and application of new therapeutic interventions
- Obstacles and facilitators to the introduction of new trauma approaches at both the individual and organisational levels.

The questionnaire was published online in a Google^©^ form from 1^st^ October until 30^th^ November 2019. The first email was sent to all Reanima ETC participants (*n* = 295) upon its inception in Portugal. After the first-round email, three reminders were sent at week 2, week 4 and week 6.

### Data analysis

Statistical analysis was conducted using the statistical software package IBM SPSS© Statistics vs. 25.0 (IBM Corp. released 2017, Armonk, NY: IBM Corp.). Descriptive statistical analysis. Categorical variables are depicted as frequencies and proportions/percentages (n and %), and quantitative variables (age and years of practice) are expressed as medians and interquartile ranges (IQRs). Missing answers for new material acquisition, implementation of new therapeutic attitudes and teamwork methodology were categorised with ‘no answer’.

Comparisons of counts for more than two categories of qualitative variables were performed using the chi-square test, and all pairwise comparisons were adjusted using Bonferroni’s correction. Comparisons were only performed for categories with sufficient counts and are presented in Tables 3 and 4. The level of significance was set at *p* < 0.05.

## Results

Out of 295 participants invited to participate in the survey, 126 (42.7%) responded. All presented percentages were calculated on the number of responses to each question.

### Respondent characteristics

Respondents were young (median (IQR) of 33 (31–36) years) and early in their careers (median (IQR) of 7 (4–10) years of practice). The majority of respondents (92/119, 77%) had no formal leadership status at their hospital, although they had a previous trauma course (67/126, 53%) and trauma experience (106/126, 84%), as well as familiarity with teaching CCM (75/126, 60%), and 94% (119/126) were still working in the trauma field. Ninety-four percent (118/126) self-reported modifications in their approach to trauma patients. These data are presented in Table 1. The majority (71/119, 60%) of respondents were, on average, involved in managing over three trauma resuscitations each month, and more than half (65/126, 52%) worked in a hospital within a multidisciplinary trauma team composed of surgeons (74/126, 64%), intensivists (62/126, 53%), anaesthesiologists (48/126, 41%), emergency medicine physicians (51/126, 43%) and nurses (106/126, 91%). The respondents’ trauma work place was: emergency room (85/119, 71%), pre-hospital care (58/119, 49%), operation room (52/119, 44%), intensive care unit (48/119, 40%) and others (12/119, 10%).

**Table 1:** Characteristics of responders (*n* = 126)

Sex (female)	76 (60%)
Age (years), Median (IQR), (*n* = 122)	33 (31–36)
Medical doctor	123 (98%)
Specialist	80 (64%)
Resident	43 (34%)
Nurse	3 (2%)
Years of practice (years), Median (IQR), (*n* = 123)	7 (4–10)
Current status in hospital, (*n* = 119)	
Formal leadership status	27 (23%)
No formal leadership status	92 (77%)
Previous Trauma Courses ^a^(Yes)	67 (53%)
Previous Experience in Teaching ^b^ (Yes)	75 (60%)
Currently ETC instructor (Yes)	31 (25%)
Previous Trauma Experience (Yes)	106 (84%)
Currently, do you deal with trauma patients? (Yes)	119 (94%)
Number of Trauma Resuscitations /month, (*n* = 119)	
<2 Trauma Resuscitations	21 (17%)
2–3 Trauma Resuscitations	27 (23%)
> 3 Trauma Resuscitations	71 (60%)
Workplace	
Hospital with Medical-Surgical emergency	65 (52%)
Trauma Centre	52 (41%)
Other places	9 (7%)
After ETC, did you modified your patient trauma approach? (Yes)	118 (94%)

^a^The majority have attended Advanced Trauma Life Support (ATLS) (27%) or Pre-hospital trauma course (9%)

^b^The majority teach Advanced Life Support (ALS) and Critical Care Medicine (CCM). Data are presented as absolute counts and (%) unless otherwise indicated. Interquartile range (IQR) is expressed as 1^st^ quartile and 3^rd^ quartile

Of those who completed the survey, 82/125 (66%) paid for the course themselves, with 35/125 (28%) having been sponsored by their employer. Eighty (68%) stated that acquiring a systematic approach to trauma patient management was the most important reason for participation in the ETC.

### The impact on clinical practice

**Fig. 1:**
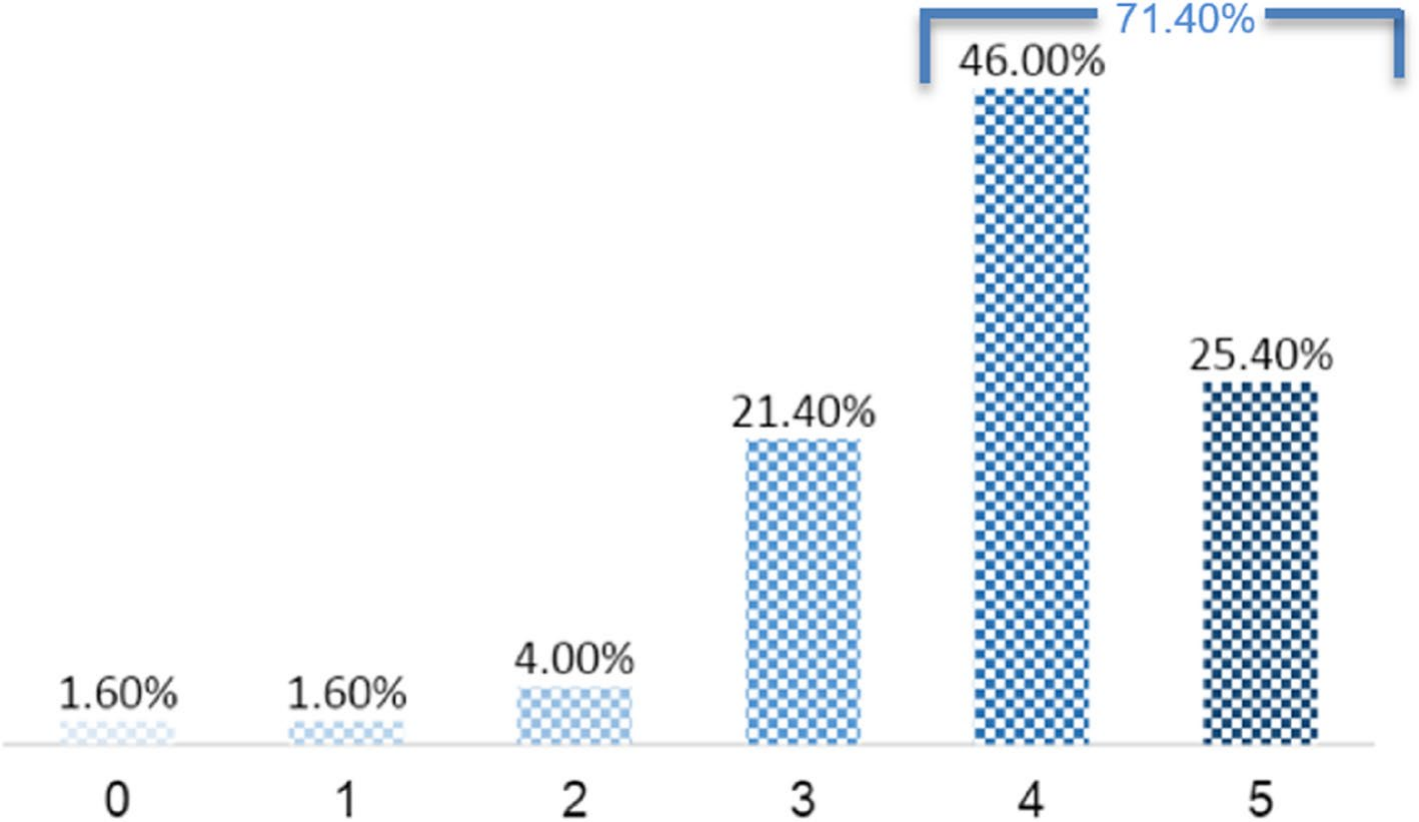
Respondents self-perceived report of the behaviour changes after participation in ETC, using a numeric scale: 0 = no behaviour changes to 5 = remarkably behaviour changes

Ninety-four percent of the respondents (118/126) affirmed that participation in the course modified their approach to trauma patients, as shown in Table 1, and 71.4% self-reported changes in their knowledge, skills and affect, which are presented in Fig. 1. Their nontechnical skills changed, particularly with regard to communication, prioritisation and teamwork, as shown in Table 2 (complete variable information in table 4, Additional file 2). When performing TTL in clinical practice, respondents without formal leadership status reported a significant change in prioritisation (71, 83.5%), and those with previous leadership experience noted significant changes in communication (26, 26.3%). Respondents who had not previously attended a trauma course identified significant changes, with more marked changes in prioritisation compared to communication skills (45, 53.6% vs. 39, 40.6%, respectively, *p* = 0.001). Those with CCM teaching experience identified significant changes in their communication skills (7, 6.8%, *p* < 0.05). When performing trauma team membership approach at workplace, respondents managing an average of less than two trauma cases per month significantly valued the structured ABCDE approach (15/72, 20.8%) and communication (16/98, 16.3%) more so than teamwork (*p* = 0.001), which is presented in Table 2.

**Table 2:** Report of respondents’ characteristics *versus* their perception of ETC impact on their clinical practice (complete variable information in Table 4, Additional file 2)

		**After attending ETC, you changed your behaviour in the initial trauma approach, Regarding Trauma Team Leadership (TTL)**			
		**Communication**	**Coordination of TM**	**Prioritising**	**Task allocation**
Current status at hospital	No formal leadership status	73_b_ (73.7%)	71_a,b_ (79.8%)	71_a_ **(83.5%)**	64_a,b_ (80.0%)
	Formal leadership status	26_a_ **(26.3%)**	18_a,b_ (20.2%)	14_b_ (16.5%)	16_a,b_ (20.0%)
Previous trauma courses	No	39_b_ (40.6%)	37_b_ (44.0%)	**45**_**a**_ **(53.6%)**	31_b_ (41.3%)
	Reanima trauma course	4_a_ **(4.2%)**	2_a,b_ (2.4%)	1_b_ (1.2%)	3_a,b_ (4.0%)
Present Critical Care Medicine teaching experience	Reanima courses	**7**_**a**_ **(6.8%)**	**3**_**b**_ **(3.4%)**	3_a,b_ (3.5%)	5_a,b_ (6.4%)
		**After attending ETC, you changed your behaviour in the initial trauma approach, regarding Trauma Team Membership (TTM)**			
		**Communication**	**Teamwork**	**Perform ABCDE safely and effectively**	
Current status at hospital	No formal leadership status	72_b_ (75.0%)	**65**_**a**_ **(83.3%)**	59_a,b_ (81.9%)	
	Formal leadership status	**24**_**a**_ **(25.0%)**	13_b_ (16.7%)	13_a,b_ (18.1%)	
Average number of Trauma Resuscitations /month	< 2 Resuscitations/month	**16**_**a**_ **(16.3%)**	7_b_ (8.9%)	**15**_**a**_ **(20.8%)**	
		**New material acquisition**			
		**None + missing**	**IO kit or needle**	**Pelvic binder**	
Sex	Female	43_b_ (53.1%)	14_a,b_ (63.6%)	27_a_ **(77.1%)**	
ETC instructor	Yes	13_b_ (16.0%)	8_a,b_ (36.4%)	17_a_ **(48.6%)**	
Average number of Trauma Resuscitations /month	< 2 Resuscitations/month	13_a_ (17.3%)	4_a_ (19.0%)	6_a_ (17.1%)	
	2 to 3 Resuscitations/month	15_a,b_ (20.0%)	2_b_ (9.5%)	11_a_ **(31.4%)**	
	> 3 Resuscitations/month	47 (62.7%)	15 (71.4%)	18 (51.4%)	
		**Implementation of new therapeutic attitudes**			
		**None + missing**	**MHP**	**Tranexamic acid**	
ETC instructor	Yes	8_b_ (14.5%)	11_a,b_ (25.6%)	21_a_ **(33.9%)**	

Data are presented as counts and percentages (n, %). Bold values represent comparisons mentioned in the text. Values in the same row not sharing the same subscript (a, b) are significantly different at *p* < 0.05 in the two-sided test of equality for column proportions. Tests adjusted for all pairwise comparisons within a row of each innermost suitable using Bonferroni’s correction. European Trauma Course (ETC), Team Member (TM), Intraosseous (IO), Massive Haemorrhage Protocol (MHP)

Being an ETC instructor (17/35, 48.6%, *p* = 0.001) and managing two to three trauma cases/month (11/35, 31.4%, *p* = 0.001) influenced the acquisition of new materials (e.g., pelvic binder) and the implementation of new therapeutic standards, namely, the use of tranexamic acid, which is presented in Table 2.

Factors affecting the transfer of knowledge in the workplace.

### At an individual level

Factors that facilitated the transfer of knowledge (no significant differences) included confidence in ETC methodology (83, 45.6%), peer support (50, 27.5%), decision-making experience (35, 19.2%) and organisational support (9, 4.9%), which are presented in Table 3 (complete variable information in table 5, Additional file 2). In contrast, lack of other ETC colleagues (28, 19.4%), low self-efficacy (27, 18.8%) and lack of motivation (26, 18.1%) all hindered knowledge transfer. Respondents with no previous trauma course experience (17, 73.9%) or no teaching experience (17, 63.0%) identified low self-efficacy as a significant obstacle for introducing new behaviour. Those with Advanced Trauma Life Support (ATLS) training (15, 60.0%) found lack of other ETC practitioners to be the main obstacle to change in the workplace, which is presented in Table 3.

**Table 3:** Report of respondents’ characteristics *versus* the factors that facilitated or hindered the transfer of learning (complete variable information in Table 5, Additional file 2)

		As an INDIVIDUAL, what FACILITATORS did you encounter in introducing new behaviours?*							
		Confidence in ETC methodology	Peer support	Decision-making experience					Institution support
All answers (% of respondents)		83 (45.6%)	50 (27.5%)	35 (19.2%)					9 (4.9%)
		**As an INDIVIDUAL, what OBSTACLES did you encounter in introducing new behaviours?**							
		None	I´m the only one with ETC training	Low self-efficacy	Lack of motivation		Other priorities		Other
All answers (% of respondents)		13 (9%)	28 (19.4%)	27 (18.8%)	26 (18.1%)		28 (19.4%)		21 (14.6%)
Previous trauma courses	No	4_a,b_ (33.3%)	**6**_**b**_** (24.0%)**	**17**_**a**_** (73.9%)**	**12**_**a,b**_** (50.0%)**		9_a,b_ (37.5%)		10_a,b_ (52.6%)
	ATLS	1_b_ (8.3%)	**15**_**a**_** (60.0%)**	**3**_**b**_** (13.0%)**	6_a,b_ (25.0%)		12_a,b_ (50.0%)		5_a,b_ (26.3%)
Previous experience in teaching	No	6_a,b_ (46.2%)	11_a,b_ (39.3%)	**17**_**a**_** (63.0%)**	9_a,b_ (34.6%)		**7**_**b**_** (25.0%)**		6_a,b_ (28.6%)
	Yes	7_a,b_ (53.8%)	17_a,b_ (60.7%)	10_b_ (37.0%)	17_a,b_ (65.4%)		**21**_**a**_** (75.0%)**		15_a,b_ (71.4%)
		**In your INSTITUTION, what FACILITATORS did you encounter in introducing new behaviours?**							
		None	Formal leadership status	Human resources availability		Material resources availability			Number of ETC professionals accredited
All answers (% of respondents)		7 (7.9%)	10 (11.2%)	21 (23.6%)		24 (27.0%)			25 (28.1%)
Previous trauma courses	No	**6**_**a**_** (85.7%)**	4_a,b_ (40.0%)	6_a,b_ (33.3%)		5_a,b_ (23.8%)			**5**_**b**_** (20.8%)**
		In your INSTITUTION, what OBSTACLES did you encounter in introducing new behaviours?*							
		Formal leadership status	Lack of human resources	Lack of knowledge about ETC methodology		Lack of material resources		Nonacceptance of the ETC methodology by colleagues	
All answers (% of respondents)		38 (17.1%)	65 (29.3%)	69 (31.1%)		30 (13.5%)		16 (7.2%)	

Data are presented as counts and percentages (n, %). * No correlations were found. Bold values represent comparisons mentioned in the text. Values in the same row not sharing the same subscript (a, b) are significantly different at *p* < 0.05 in the two-sided test of equality for column proportions. Tests adjusted for all pairwise comparisons within a row of each innermost suitable using Bonferroni’s correction. European Trauma Course (ETC)

### At an organisational level

Change in organisational behaviour was linked to the number of ETC-accredited staff (25, 28.1%), material resource availability (24, 27.0%) and human resources (21, 23.6%), although without statistical significance, which are presented in Table 3. No significant differences were observed for the identification of obstacles in introducing new behaviours.

## Discussion

This study provides evidence that participation in the ETC can be one of the elements that can lead to individual behavioural changes in the workplace. However, the ability to influence others and bring about institutional or regional organisational changes was more difficult to achieve. Major factors were the status of the person at the workplace, their previous experience (trauma clinical practice and coursework, as well as in teaching CCM) and self-efficacy. Being an ETC instructor influenced the acquisition of new material and the implementation of new therapeutic attitudes. The Reanima ETC survey identified factors that facilitate or hinder the transfer of experiential learning at the individual, organisational and national levels.

To the best of our knowledge, this is the first study focusing on the ETC impact on candidates’ professional behaviour towards their management of major trauma. A search of the biomedical and medical education databases produced no mentions of the ETC other than those articles written by members on the early development of the ETC group [1–4]. Over the last three decades, there has been an exponential increase in the number of life-support type courses. Examples include Advanced Cardiac Life Support (ACLS) and ATLS, among others. Although popular and apparently well-received, evidence for their impact in the workplace is heterogeneous [13–15]. Hattie et al. (2013) hypothesised that almost any approach to learning might work, but later in 2021, they also identified through a meta-analysis ten factors that effectively influence the process of learning [16, 17]. Despite a paucity of data on the effect of ATLS on trauma mortality, existing evidence supports its practice as a means of decreasing mortality and improving systems of care globally [18–20].

To date, the assessment of the ETC, organised by Reanima, has been based on postcourse evaluations by candidates, national faculty and audits by international faculty. For some evaluations, Kirkpatrick model is applied mainly at level 1.

Reaction, also called “customer satisfaction”, i.e., how participants perceived the training; and level 2. Learning, i.e., at what level participants acquire more knowledge, new behaviours, and values and develop skills as a result of their participation in the course. All feedback has been very positive, which encouraged us as an ETC organiser to proceed. The scarcity of evidence on the impact on trainees’ clinical practice challenged us to clarify whether trainees applied the newly acquired ETC knowledge and changed their behaviour in their workplace. Recently, Greif et al. pointed out the importance of ‘educational efficiency’, arguing that educational approaches are critical links between scientific findings and their implementation into practice [21, 22]. A questionnaire based on Holton’s evaluation model [6, 12] was used to assess whether the ETC leads to behavioural changes in the workplace, i.e. if occurred knowledge transfer to the workplace. Our survey results were very promising, with 94% of the respondents reporting that the ETC modified their approach to trauma patients, as shown in Table 1, and 71.4% significantly changed their behaviour, as presented in Fig. 1. These data lead us to infer that ETC methodology, providing a robust educational approach, could be one of the factors favouring knowledge transfer to practice, meeting what 68% of the respondents considered the most important reason for participation in the ETC, i.e., acquiring a systematic approach to trauma patient management.

Research indicates that teamwork and leadership are increasingly recognised as important factors contributing to patient safety and outcomes in health care [23–25]. These are important goals of the ETC methodology, where NTS are developed to improve how technical skills, tasks and procedures are carried out by trainees [26]. Post ETC respondents acknowledged behaviour changes regarding the NTS, such as communication, prioritisation and teamwork, which are presented in Table 2. The changes were dependent on formal leadership status at the hospital, previous training in trauma courses, trauma experience and experience with teaching CCM. Respondents without leadership status registered changes in prioritising tasks, acquiring the team leadership skills emphasised in the ETC. In contrast, respondents with leadership status noted communication as the main area of change. What was considered most important varied with the respondent’s current status and frequency of trauma cases managed per month.

ETC instructors held a strategic position as influencers for the acquisition of new institutional materials (e.g., pelvic binder) and implementation of new therapeutic standards in massive haemorrhage (e.g., standard use of tranexamic acid). These results point out the opportunity and ability of instructors to change their workplace.

There are two main aspects of knowledge transfer: the underlying process and the factors that facilitate or hinder it [12]. Reanima ETC study findings have helped to clarify some factors that influence the transfer of learning [27–30].

At an individual level, confidence in ETC methodology and peer support were the most common facilitators for introducing new behaviour changes, as presented in Table 3. The obstacles to changing behaviour were personality characteristics (for instance, some recognised themselves as lacking self-efficacy, especially those with no previous trauma coursework or teaching experience) and other priorities related to career choices. Respondents with ATLS training found a lack of ETC practitioners in their place of work to be the main obstacle to change, indicating a need for more ETC courses.

A change in organisational behaviour was linked to the number of ETC-accredited professionals and the availability of material and human resources, which is not surprising.

However, the majority of trainees (92, 77% of the 119 that answered the question) had no formal leadership status at their hospital, which could be a reason why we observed fewer dramatic regional organisational changes. Other obstacles to introducing new behaviours included lack of knowledge about ETC methodology by other professionals (69, 31.1%) and limited human resources (65, 29.3%), as presented in Table 3. Again, these findings emphasise the need for ETC dissemination. As ETC has only recently been implemented in Portugal, it would be worth reassessing this in the future when there is a greater share of experiences and knowledge.

Although the minority of respondents had a formal leadership status at their hospital, the positive impact of the ETC has led the Portuguese College of Anaesthesiology to integrate ETC into their trainees’ resident program. This follows their evaluation of it being a good educational model for improving trauma resuscitation training. A further national impact of the ETC has been the prioritisation of the National Trauma Registry, which aims to provide a useful tool in the quality evaluation and improvement of trauma care [31].

Our survey has inherent limitations. First, selection bias cannot be ignored. Given the process of dissemination, it is difficult to provide an estimation of the nonrespondent-to-respondent ratio. Sample size calculations require assumptions about expected event in different groups or, upon expected effect sizes. Therefore, when no guesstimates or expectations are possible (if no assumptions can be made), and no pilot studies were conducted previously, a sample of 126 respondents (which is not large) might be considered reasonable (> 40%) for the field when considering the target population size (*N* = 295) [32, 33].

Furthermore, any self-report survey is prone to bias; thus, we could not assess the gap between routine practice and self-perception. As such, attitude change in the present study reflects no more than self-perceived and self-reported change.

Effective implementation of a science field, namely trauma education, needs the application of efficient and evidence‐based interventions to improve the health and well‐being of trauma population. It involves: diffusion, dissemination, implementation, adoption and sustainability [34–38].

## Conclusion

The investigators acknowledge that ETC can be one of the elements that has impact on Reanima trainees´ recognizing the changes introduced and adopted in their own clinical practice, in their work place and in the end, in the political authorities who manage national healthcare systems. This is highlighted by their behaviour change as they had acquired new knowledge, values, attitudes and skills (practical and procedural). They were able to introduce change into their organizations, disseminating knowledge, i.e., the implementation and adoption of ETC trauma approach. Further improvement to obtain sustainability of ETC project and improve the health and well‐being of trauma population may also be possible through international trauma registry development that will guide us to other achievements, such as the impact of the ETC trauma approach on patient outcomes.

The Reanima ETC survey delivers several key messages. The combination of highly motivated trainees with the ETC educational efficiency methodology was linked with a changing approach to trauma. However, the positive responses from the individual candidates do not automatically translate into organisational changes. Other confounding factors can help or hinder this transformation. Ultimately, both the individual and the organisation need to be in synchrony so that the full impact on the trauma patient’s outcome can be achieved and measured. This is a topic for a future study.

## Supplementary Information

**Additional file 1.**

**Additional file 2: Table 4.** Report of respondents’ characteristics versus their perception of ETC impact on their clinical practice (complete variables’ information). **Table 5.** Report of respondents’ characteristics versus the factors that facilitated or hindered the transfer of learning (complete variables’ information).

## Data Availability

The datasets used and analysed during the current study are available from the corresponding author upon reasonable request. Supplementary material related to this article can be found in attachment form 1.

## Abbreviations

ETC: European Trauma Course
CCM: Critical Care Medicine
ACLS: Advanced Cardiac Life Support
ATLS: Advanced Trauma Life Support
NTS: Non-Technical Skills
TTL: Trauma Team Leader
TTM: Trauma Team Member
TEAM: Trauma Evaluation and Management course
