# Supplementary material for: Does participation in the European Trauma Course lead to new behaviours and organisational change? A Portuguese experience
Source: BMC Med Educ. 2023 Jun 6;23:415. doi: 10.1186/s12909-023-04322-0 (PMC10245415; doi:10.1186/s12909-023-04322-0)
Supplement: Supplementary file 1 — Additional file 1. [file 12909_2023_4322_MOESM1_ESM.docx]

Additional file 1

**REANIMA EUROPEAN TRAUMA COURSE R-ETC) EVALUATION QUESTIONNAIRE**

**SECTION I: RESPONDENTS BACKGROUND**

**1. Sex** (Drop down menu - single choice)

- Female
- Male

**2. Age** (Number)

**3. In which region do you practice?** (Drop down menu- single choice)

- North
- Center
- South
- Azores
- Other

**4. What is the classification of your hospital?** (Drop down menu- single choice)

- Hospital with Medical and Surgical emergency and Trauma Center
- Hospital with Medical and Surgical emergency
- Hospital with Basic emergency
- Local Health Unit
- Pre-hospital Service

**5. What is your professional category?** (Drop down menu- single choice)

- Medical doctor
- Nurse
- Other

**6. What is your main specialty?** (Drop down menu- single choice)

- Intensive Care Medicine
- Anesthesiology
- Emergency Medicine
- Internal Medicine
- General Surgery
- Neurosurgery
- Orthopedic Surgery
- General Practitioner
- Other

**7. For how many years have you been practicing?** (Number)

**8. Now, what position do you hold?** (Drop down menu- single choice)

- Formal leadership status
- No formal leadership status

**9. What was your motivation to do ETC?** (Drop down menu- multiple choice)

- Acquire a systematic approach to trauma patient
- Integrated in Reanima Medical Post- Graduation
- Professional need
- Personal interest in trauma
- Introduce ETC methodology in Portugal
- Observe new methodologies from ETC
- Other

**10. Course payment?** (Drop down menu- single choice)

- Paid by self
- Sponsored by employer
- Sponsored by Reanima
- Other

**11. Did you have previous trauma courses?** (Drop down Menu- single choice)

- Yes
- No

**12. What trauma courses do you have previous ETC?** (Drop down menu- multiple choice)

- Advanced Trauma Life Support (ATLS)
- Prehospital Trauma Life Support (PHTLS)
- International Trauma Life Support (ITLS)
- Suporte Vital Avançado Trauma (SVAT)
- Trauma Evaluation and Management (TEAM)- American College of Surgeons
- Pre-hospital Trauma Course
- Medical Reanima Trauma Course
- Nurse Reanima Trauma Course
- Other

**13. Did you have pre-ETC trauma experience?** (Drop down menu- single choice)

- Yes
- No

**14. Did you have previous experience in teaching?** (Drop down menu- single choice)

- Yes
- No

**15. Do you have present experience area in teaching?** (Drop down Menu- multiple choice)

- Advanced Life Support (ALS)
- Immediate Life Support (ILS)
- Basic Life Support (BLS)
- European Paediatric Advanced Life Support (EPALS)
- Winfocus instructor
- Reanima sepsis course
- Loco regional anesthesia course
- National Institute of Medical Emergency (INEM) courses
- Fundamental Critical Care Support Courses (FCCS)
- Ventilation courses
- University teaching
- None
- Other

**SECTION II: ETC DATA**

**1. What was the year of your ETC?** (Number)

**2. You were nominated as an IP?** (Drop down menu- single choice)

- Yes
- No

**3. Now, are you an ETC instructor?** (Drop down menu- single choice)

- Yes
- No

**4. For how long are you an ETC instructor?** (Number)

**SECTION III: TRAUMA CONTEXT DAILY**

1. **Do you deal with trauma situations on your daily life?** (Drop down menu- single choice)

- Yes
- No

1. **How many trauma situations do you deal per month (average)?**

(Drop down Menu- single choice)

- >3
- 2 to 3
- <2

1. **At what context do you deal with trauma situations?** (Drop down Menu- single choice)

- Pre-hospital care
- Emergency room
- Operating Room
- Intensive Care Unit
- Intermediate Care Unit
- Transport Unit of Azores
- Other

**4. Currently what title do you hold?** (Drop down Menu- single choice)

- Specialist doctor
- Specialist nurse
- Resident

1. **What was the motivation to do an ETC course:** (Drop down menu - multiple choice)

- Acquire a systematic approach to trauma patient
- Professional need
- Personal interest in trauma
- Reanima post-graduation
- Introduce ETC methodology in Portugal
- None
- Other

**SECTION IV: ETC IMPACT ON TRAINEE’S PRACTICE**

1. **After attending ETC, you changed your behaviour in the initial trauma approach, regarding Team Leadership:** (Drop down menu- multiple choice)

- Task allocation
- Coordination the Team Members
- Prioritizing
- Communication
- None
- Other

1. **After attending ETC, you changed your behaviour in the initial trauma approach, regarding Team Membership:** (Drop down menu- multiple choice)

- Cooperation with other elements
- Perform ABCDE safely and effectively
- Communication
- None
- Other

1. **After attending ETC, you changed your behaviour in the initial trauma approach, regarding communication with professionals and family?**

(Drop down menu- single choice)

- Yes
- No

1. **Did ETC contributed to modify the initial trauma approach, in your work context?** (Drop down menu- single choice)

- Yes
- No

1. **Number of trauma team members?** (Drop down menu- single choice)

- >3
- 2 to 3
- <2
- Other

1. **Professionals involved in the trauma team** (Drop down menu- multiple choice)

- Anesthesiology
- Emergency physicians (Emergency Medicine)
- Intensive Care Medicine (Intensivists)
- Internal Medicine
- General Surgery
- Neurosurgery
- Orthopedic Surgery
- Nurses
- Operational assistants
- Other

1. **New material acquisition?** (Drop down Menu- multiple choice)

- Pelvic binder
- Intra Osseous kit or needle
- None
- Other

1. **Implementation of new therapeutic attitudes?** (Drop down menu- multiple choice)

- Tranexamic acid
- Massive Hemorrhage Protocol (MHP)
- None
- Other

1. **ETC Teamwork methodology** (Drop down menu- multiple choice)

- Horizontal approach
- Planning
- Communication with team, patients and family
- Safe patient transport
- Debriefing
- None
- Other

1. **As an individual, what obstacles did you encounter in introducing new behaviours**

(Drop down menu- multiple choice)

- I´m the only one with ETC training
- Self-efficacy
- Lack of motivation
- Other priorities
- Formal leadership status
- None
- Other

1. **In your institution, what obstacles did you encounter in introducing new behaviours** (Drop down menu- multiple choice)

- Lack of material resources
- Lack of human resources
- Lack of knowledge about ETC methodology
- Non-acceptance of the ETC methodology by colleagues
- Formal leadership status
- None
- Other

1. **As an individual, what facilitators did you encounter in introducing new behaviours** (Drop down Menu- multiple choice)

- Confidence in ETC methodology
- Power to decide implementation
- Peer support
- Institution support
- None
- Other

1. **In your institution, what facilitators did you encounter in introducing new behaviours** (Drop down Menu- multiple choice)

- Number of ETC professionals accredited
- Human resources availability
- Material resources availability
- Formal leadership status
- None
- Other

1. **Did participation in ETC led to a modification of your behaviour in reality**

(Drop down menu- single choice)

- Yes
- No

**15. Set at what extent to which participation in ETC led to a modification of your behaviour using a numeric scale from zero to five** (Drop down menu- single choice)

- **0** (= not at all)
- **1**
- **2**
- **3**
- **4**
- **5** (= extremely)

**SECTION V: COMMENTS AND SUGGESTIONS**

1. Open field
2. **Auxiliary: Do you want to leave your email and receive the results of the survey?**
