# Supplementary material for: Does participation in the European Trauma Course lead to new behaviours and organisational change? A Portuguese experience
Source: BMC Med Educ. 2023 Jun 6;23:415. doi: 10.1186/s12909-023-04322-0 (PMC10245415; doi:10.1186/s12909-023-04322-0)
Supplement: Supplementary file 2 — Additional file 2: Table 4. Report of respondents’ characteristics versus their perception of ETC impact on their clinical practice (complete variables’ information). Table 5. Report of respondents’ characteristics versus the factors that facilitated or hindered the transfer of learning (complete variables’ information). [file 12909_2023_4322_MOESM2_ESM.docx]

**Additional file 2**

**Table 4 –** Report of respondents’ characteristics *versus* their perception of ETC impact on their clinical practice (complete variables’ information)

|  | | After attending ETC, you changed your behaviour in the initial trauma approach,  Regarding Trauma Team Leadership (TTL) | | | | | | | | | | | | |
| --- | --- | --- | --- | --- | --- | --- | --- | --- | --- | --- | --- | --- | --- | --- |
|  |  | None | Communication | Coordination of TM | | | Prioritising | | Task allocation | | | | Others | |
| Current status in hospital | No formal leadership status | 3 (100.0%) | 73_b_ (73.7%) | 71_a,b_ (79.8%) | | | 71_a_ **(83.5%)** | | 64_a,b_ (80.0%) | | | | 2 (66.7%) | |
|  | Formal leadership status | 0 (0.0%) | 26_a_ **(26.3%)** | 18_a,b_ (20.2%) | | | 14_b_ (16.5%) | | 16_a,b_ (20.0%) | | | | 1 (33.3%) | |
| Previous trauma courses | No | 1 (33.3%) | 39_b_ (40.6%) | 37_b_ (44.0%) | | | **45_a_ (53.6%)** | | 31_b_ (41.3%) | | | | 1 (33.3%) | |
|  | ATLS | 1 (33.3%) | 36 (37.5%) | 30 (35.7%) | | | 27 (32.1%) | | 29 (38.7%) | | | | 1 (33.3%) | |
|  | Prehospital trauma course | 0 (0.0%) | 18 (18.8%) | 15 (17.9%) | | | 11 (13.1%) | | 13 (17.3%) | | | | 0 (0.0%) | |
|  | Reanima trauma course | 1 (33.3%) | 4_a_ **(4.2%)** | 2_a,b_ (2.4%) | | | 1_b_ (1.2%) | | 3_a,b_ (4.0%) | | | | 1 (33.3%) | |
|  | other: SVAT, PHTLS, ITLS, DATC, TEAM | 0 (0.0%) | 8 (8.3%) | 8 (9.5%) | | | 7 (8.3%) | | 6 (8.0%) | | | | 0 (0.0%) | |
| Present experience Area in teaching | No | 2 (66.7%) | 37 (35.9%) | 32 (36.4%) | | | 36 (41.9%) | | 27 (34.6%) | | | | 0 (0.0%) | |
|  | Reanimation courses | 1 (33.3%) | 33 (32.0%) | 28 (31.8%) | | | 24 (27.9%) | | 26 (33.3%) | | | | 1 (33.3%) | |
|  | Emergency and critical care | 1 (33.3%) | 19 (18.4%) | 18 (20.5%) | | | 18 (20.9%) | | 13 (16.7%) | | | | 2 (66.7%) | |
|  | Trauma courses | 0 (0.0%) | 11 (10.7%) | 10 (11.4%) | | | 9 (10.5%) | | 9 (11.5%) | | | | 0 (0.0%) | |
|  | Post-Graduated teaching | 0 (0.0%) | 2 (1.9%) | 1 (1.1%) | | | 1 (1.2%) | | 2 (2.6%) | | | | 1 (33.3%) | |
|  | Pre-Graduated teaching | 0 (0.0%) | 15 (14.6%) | 13 (14.8%) | | | 12 (14.0%) | | 14 (17.9%) | | | | 0 (0.0%) | |
|  | Reanima courses | 0 (0.0%) | **7_a_ (6.8%)** | **3_b_ (3.4%)** | | | 3_a,b_ (3.5%) | | 5_a,b_ (6.4%) | | | | 0 (0.0%) | |
|  | Simulation | 0 (0.0%) | 1 (1.0%) | 1 (1.1%) | | | 1 (1.2%) | | 1 (1.3%) | | | | 0 (0.0%) | |
|  | VMER experience | 0 (0.0%) | 1 (1.0%) | 1 (1.1%) | | | 1 (1.2%) | | 0 (0.0%) | | | | 0 (0.0%) | |
|  | Other | 0 (0.0%) | 2 (1.9%) | 2 (2.3%) | | | 2 (2.3%) | | 2 (2.6%) | | | | 00.0% | |
|  | | After attending ETC, you changed your behaviour in the initial trauma approach,  regarding Trauma Team Membership (TTM) | | | | | | | | | | | | |
|  |  | None | Communication | Teamwork | | | | Perform ABCDE safely and effectively | | | | | Others | |
| Current status in hospital | No formal leadership status | 3 (75.0%) | 72_b_ (75.0%) | **65_a_ (83.3%)** | | | | 59_a,b_ (81.9%) | | | | | 1 (33.3%) | |
|  | Formal leadership status | 1 (25.0%) | **24_a_ (25.0%)** | 13_b_ (16.7%) | | | | 13_a,b_ (18.1%) | | | | | 2 (66.7%) | |
| Average number of Trauma Resuscitations /month | <2 Resuscitations/month | 2 (50.0%) | **16_a_ (16.3%)** | 7_b_ (8.9%) | | | | **15_a_ (20.8%)** | | | | | 0 (0.0%) | |
|  | 2 to 3 Resuscitations/month | 0 (0.0%) | 24 (24.5%) | 22 (27.8%) | | | | 14 (19.4%) | | | | | 1 (33.3%) | |
|  | >3 Resuscitations/month | 2 (50.0%) | 58 (59.2%) | 50 (63.3%) | | | | 43 (59.7%) | | | | | 2 (66.7%) | |
|  | | New material acquisition | | | | | | | | | | | | |
|  |  | None+ missing | | | IO kit or needle | | | | | | | Pelvic binder | | |
| Sex | Female | 43_b_ (53.1%) | | | 14_a,b_ (63.6%) | | | | | | 27_a_ **(77.1%)** | | | |
|  | Male | 38_a_ (46.9%) | | | 8_a,b_ (36.4%) | | | | | | 8_b_ (22.9%) | | | |
| ETC instructor | No | 68_a_ (84.0%) | | | 14_a,b_ (63.6%) | | | | | | 18_b_ (51.4%) | | | |
|  | Yes | 13_b_ (16.0%) | | | 8_a,b_ (36.4%) | | | | | | 17_a_ **(48.6%)** | | | |
| Average number of Trauma Resuscitations /month | <2 Resuscitations /month | 13_a_ (17.3%) | | | 4_a_ (19.0%) | | | | | | 6_a_ (17.1%) | | | |
|  | 2 to 3 Resuscitations /month | 15_a,b_ (20.0%) | | | 2_b_ (9.5%) | | | | | | 11_a_ **(31.4%)** | | | |
|  | >3 Resuscitations /month | 47 (62.7%) | | | 15 (71.4%) | | | | | | 18 (51.4%) | | | |
|  | | Implementation of new therapeutic attitudes | | | | | | | | | | | | |
|  |  | None+ missing | | | MHP | | | | | | Tranexamic acid | | | |
| ETC instructor | No | 47_a_ **(85.5%)** | | | 32_a,b_ (74.4%) | | | | | | 41_b_ (66.1%) | | | |
|  | Yes | 8_b_ (14.5%) | | | 11_a,b_ (25.6%) | | | | | | 21_a_ **(33.9%)** | | | |
|  | | Teamwork methodology | | | | | | | | | | | | |
|  |  | NAP+ missing | Horizontal approach | Planning | | Communication with team, patients and family | | | | Safe patient transport | | | | Debriefing |
| Previous trauma courses | No | 7 (43.8%) | 21 (41.2%) | 31 (43.1%) | | 29 (38.7%) | | | | 28 (47.5%) | | | | 22 (40.0%) |
|  | ATLS | 5_a,b_ (31.3%) | 18_a,b_ (35.3%) | **29_a_ (40.3%)** | | 27_a,b_ (36.0%) | | | | **16_b_ (27.1%)** | | | | 23_a,b_ (41.8%) |
|  | Prehospital trauma course | 1 (6.3%) | 11 (21.6%) | 11 (15.3%) | | 14 (18.7%) | | | | 10 (16.9%) | | | | 11 (20.0%) |
|  | Medical and Nurse Reanima trauma course | 2 (12.5%) | 1 (2.0%) | 3 (4.2%) | | 4 (5.3%) | | | | 2 (3.4%) | | | | 1 (1.8%) |
|  | other: SVAT, PHTLS, ITLS, DATC, TEAM | 1 (6.3%) | 5 (9.8%) | 6 (8.3%) | | 6 (8.0%) | | | | 6 (10.2%) | | | | 6 (10.9%) |

Note: Data are presented as counts and percentages (n, %). NAP- Not applicable. Bold values represent comparisons mentioned in the text. Values in the same row not sharing the same subscript (a, b) are significantly different at p< 0.05 in the two-sided test of equality for column proportions. Tests adjusted for all pairwise comparisons within a row of each innermost suitable using Bonferroni’s correction. Grey highlighted columns show data that were not compared (due to low counts), Suporte Vital Avançado en Trauma (SVAT), Pre-Hospital Trauma Life Support (PHTLS), International Trauma Life Support (ITLS), Definitive Anaesthetic Trauma Care (DATC), Trauma Evaluation and Management course (TEAM). European Trauma Course (ETC), Team Member (TM), Intraosseous (IO), Massive Haemorrhage Protocol (MHP).

**Table 5-** Report of respondents’ characteristics *versus* the factors that facilitated or hindered the transfer of learning (complete variables’ information)

|  | | As an INDIVIDUAL, what FACILITATORS did you encounter in introducing new behaviours?* | | | | | | | | | | | | | | | | | | | |
| --- | --- | --- | --- | --- | --- | --- | --- | --- | --- | --- | --- | --- | --- | --- | --- | --- | --- | --- | --- | --- | --- |
|  |  | None | | | Confidence in ETC methodology | | | Peer  support | | | | Power to decide implementation | | | | | Institution support | | Motivation | | |
| All answers (% of respondents) | | 4 (2.2%) | | | 83 (45.6%) | | | 50 (27.5%) | | | | 35 (19.2%) | | | | | 9 (4.9%) | | 1 (0.5%) | | |
|  | | As an INDIVIDUAL, what OBSTACLES did you encounter in introducing new behaviours? | | | | | | | | | | | | | | | | | | | |
|  |  | None | | I´m the only one with ETC training | | | Lack of self-efficacy | | | Lack of motivation | | | | Other priorities | | | | Formal leadership status | | Other | |
| All answers (% of respondents) | | 13 (9%) | | 28 (19.4%) | | | 27 (18.8%) | | | 26 (18.1%) | | | | 28 (19.4%) | | | | 1 (0.7%) | | 21 (14.6%) | |
| Previous trauma courses | No | 4_a,b_ (33.3%) | | **6_b_ (24.0%)** | | | **17_a_ (73.9%)** | | | 12_a,b_ (50.0%) | | | | 9_a,b_ (37.5%) | | | | 1 (100%) | | 10_a,b_ (52.6%) | |
|  | ATLS | 1_b_ (8.3%) | | **15_a_ (60.0%)** | | | **3_b_ (13.0%)** | | | 6_a,b_ (25.0%) | | | | 12_a,b_ (50.0%) | | | | 0 (0.0%) | | 5_a,b_ (26.3%) | |
|  | Prehospital trauma course | 5 (41.7%) | | 4 (16.0%) | | | 2 (8.7%) | | | 5 (20.8%) | | | | 3 (12.5%) | | | | 0 (0.0%) | | 4 (21.1%) | |
|  | Medical and Nurse Reanima trauma course | 3 (25.0%) | | 0 (0.0%) | | | 1 (4.3%) | | | 1 (4.2%) | | | | 1 (4.2%) | | | | 0 (0.0%) | | 1 (5.3%) | |
|  | other: SVAT, PHTLS, ITLS, DATC, TEAM | 0 (0.0%) | | 5 (20.0%) | | | 1 (4.3%) | | | 2 (8.3%) | | | | 1 (4.2%) | | | | 0 (0.0%) | | 2 (10.5%) | |
| Previous experience in teaching | No | 6_a,b_ (46.2%) | | 11_a,b_ (39.3%) | | | **17_a_ (63.0%)** | | | 9_a,b_ (34.6%) | | | | **7_b_ (25.0%)** | | | | 1 (100%) | | 6_a,b_ (28.6%) | |
|  | Yes | 7_a,b_ (53.8%) | | 17_a,b_ (60.7%) | | | 10_b_ (37.0%) | | | 17_a,b_ (65.4%) | | | | **21_a_ (75.0%)** | | | | 0(0.0%) | | 15_a,b_ (71.4%) | |
|  | | In your INSTITUTION, what FACILITATORS did you encounter in introducing new behaviours | | | | | | | | | | | | | | | | | | | |
|  |  | None | Formal leadership status | | | Human resources availability | | | | | Material resources availability | | | | Number of ETC professionals accredited | | | | | Other | |
| All answers (% of respondents) | | 7 (7.9%) | 10 (11.2%) | | | 21 (23.6%) | | | | | 24 (27.0%) | | | | 25 (28.1%) | | | | | 2 (2.2%) | |
| Previous trauma courses | No | **6_a_ (85.7%)** | 4_a,b_ (40.0%) | | | 6_a,b_ (33.3%) | | | | | 5_a,b_ (23.8%) | | | | **5_b_ (20.8%)** | | | | | 1 (50.0%) | |
|  | ATLS | 1 (14.3%) | 5 (50.0%) | | | 11 (61.1%) | | | | | 14 (66.7%) | | | | 12 (50.0%) | | | | | 0 (0.0%) | |
|  | Prehospital trauma course | 0 (0.0%) | 0 (0.0%) | | | 2 (11.1%) | | | | | 2 (9.5%) | | | | 6 (25.0%) | | | | | 0 (0.0%) | |
|  | Medical and Nurse Reanima trauma course | 0 (0.0%) | 0 (0.0%) | | | 1 (5.6%) | | | | | 1 (4.8%) | | | | 3 (12.5%) | | | | | 0 (0.0%) | |
|  | other: SVAT, PHTLS, ITLS, DATC, TEAM | 0 (0.0%) | 110.0% | | | 0 (0.0%) | | | | | 1 (4.8%) | | | | 1 (4.2%) | | | | | 1 (50.0%) | |
|  | | In your INSTITUTION, what OBSTACLES did you encounter in introducing new behaviours* | | | | | | | | | | | | | | | | | | | |
|  |  | None (NAP) | Formal leadership status | | | Lack of human resources | | | Lack of knowledge about ETC methodology | | | | Lack of material resources | | | Nonacceptance of the ETC methodology by colleagues | | | | | Other |
| All answers (% of respondents) | | 2 (0.9%) | 38 (17.1%) | | | 65 (29.3%) | | | 69 (31.1%) | | | | 30 (13.5%) | | | 16 (7.2%) | | | | | 2 (0.9%) |

Note: Data are presented as counts and percentages (n, %). * No correlations were found. Bold values represent comparisons mentioned in the text. Values in the same row not sharing the same subscript (a, b) are significantly different at p< 0.05 in the two-sided test of equality for column proportions. Tests adjusted for all pairwise comparisons within a row of each innermost suitable using Bonferroni’s correction. Grey highlighted columns show data that were not compared (due to low counts).
